# State-level drivers of future fine particulate matter mortality in the United States

**DOI:** 10.1088/1748-9326/ab59cb

**Published:** 2019-12-18

**Authors:** Yang Ou, Steven J Smith, J Jason West, Christopher G Nolte, Daniel H Loughlin

**Affiliations:** 1 Oak Ridge Institute for Science and Education, United States of America; 2 Center for Environmental Measurement and Modeling, US Environmental Protection Agency, RTP, NC, United States of America; 3 Environmental Sciences and Engineering, University of North Carolina at Chapel Hill, United States of America; 4 Joint Global Change Research Institute, Pacific Northwest National Laboratory, College Park, MD, United States of America

**Keywords:** energy, air quality management, health impacts, human-earth system model, air pollution, emission projection

## Abstract

Future fine particulate matter (PM_2_._5_) concentrations and resulting health impacts will be largely determined by factors such as energy use, fuel choices, emission controls, state and national policies, and demographcs. In this study, a human-earth system model is used to estimate PM_2.5_ mortality costs (PMMC) due to air pollutant emissions from each US state over the period 2015 to 2050, considering current major air quality and energy regulations. Contributions of various socioeconomic and energy factors to PMMC are quantified using the Logarithmic Mean Divisia Index. National PMMC are estimated to decrease 25% from 2015 to 2050, driven by decreases in energy intensity and PMMC per unit consumption of electric sector coal and transportation liquids. These factors together contribute 68% of the decrease, primarily from technology improvements and air quality regulations. States with greater population and economic growth, but with fewer clean energy resources, are more likely to face significant challenges in reducing future PMMC from their emissions. In contrast, states with larger projected decreases in PMMC have smaller increases in population and per capita GDP, and greater decreases in electric sector coal share and PMMC per unit fuel consumption.

## Introduction and objectives

Long term trends in air quality and health burdens will be driven by pollutant emissions and underlying socioeconomic factors. Emissions sources such as factories, motor vehicles, and electric utilities contribute to total concentrations of PM_2.5_ by emitting PM_2.5_ and its precursors, including nitrogen oxides (NO_*x*_), sulfur dioxide (SO_2_), and ammonia (NH_3_). Over recent decades, regulatory efforts targeting these major emission sources have substantially reduced the US public health burden from air pollution (Butt *et al* 2017, Fann *et al* 2018, Shapiro and Walker 2018, Zhang *et al* 2018). Previous studies have applied chemical transport models (CTMs) to attribute total PM_2_._5_ health impacts to specific emission sources and locations (Fann *et al* 2012, Caiazzo *et al* 2013, Dedoussi and Barrett 2014, Turner *et al* 2015, Penn *et al* 2017).

These studies generally found that PM_2.5_ health impacts arise from many different emission sources (Turner *et al* 2015) that affect public health by their proximity to population exposed (Fann *et al* 2009). For example, most electric sector PM_2_._5_ health impacts are attributable to emissions from states that have considerable coal-fired electricity production and large downwind population, while health impacts of the residential sector are mainly attributed to states with large population and significant residential wood combustion (Penn *et al* 2017).

A variety of socioeconomic factors also affect future changes in PM_2.5_ health impacts. First, socioeconomic development increases service and energy demands (Riahi *et al* 2017), potentially resulting in greater fuel combustion. Second, population growth not only boosts energy demand but also typically increases population exposure to air pollution. Third, the challenges and opportunities for reducing adverse PM_2_._5_ health impacts may differ considerably among states, depending on historical energy use patterns (Brown *et al* 2018), and regional policies (Shi *et al* 2017), such as the cross-state air pollution rule (CSAPR) (EPA 2011a) which caps electric sector emissions of NO_*x*_ and SO_2_ for 23 states.

Several other studies have employed reduced-form models based on CTM simulations to estimate the PM_2_._5_ health benefits of reducing a tonne of pollutant emission (deaths/tonne or, considering monetized mortality, dollars/tonne) across different sectors, pollutant species, or years (Fann *et al* 2009, Fann *et al* 2012, Muller 2014, Goodkind *et al* 2019). Despite the insights gained from these studies, they are generally based on a single year’s emission conditions, without considering changes due to the socioeconomic factors mentioned previously.

Therefore, it remains unclear how future changes in polluting sources and socioeconomic factors will jointly affect future PM_2.5_ health impacts in the United States. Anticipating the effects of these factors for each state could be important from a public health perspective and support the development of long-term air quality management strategies. In this study, we aim to (1) project the change in monetized PM_2.5_ mortality costs (hereinafter referred to as ‘PMMC’), a metric commonly used to represent the health impact of air pollution, from 2015 to 2050 at the US national and state levels under current regulations; (2) quantify the relative influences of socioeconomic and energy factors driving these changes for each state, and (3) identify the PMMC drivers that lead to different trends among states.

To achieve these objectives, a human-earth system model with US state-level resolution is used to project future state-level air pollutant emissions, considering factors such as population growth and migration, economic growth, technology change, resource availability, and energy and emissions policies. Year-, pollutant-, sector-, and state-specific PMMC coefficients (dollars/tonne, Heo *et al* 2016a) are integrated into this framework to estimate PMMC from 2015 to 2050. We then quantify the contribution of socioeconomic, energy, and emission-related factors to PMMC changes for each state. To our knowledge, this paper presents the first comprehensive evaluation of the relative importance of concurrent socioeconomic and energy drivers to changes in future PMMC for individual states.

## Methods

### Research tool and scenario design

The global change assessment model (GCAM) is a partial equilibrium human-earth system model that represents the linkages between global energy, water, land, climate, and economic systems (Calvin *et al* 2019, JGCRI 2019a). In GCAM, exogenous population, GDP, and labor productivity assumptions drive overall energy and service demands, and a logit function (McFadden 1973, Clarke and Edmonds 1993) is used to estimate the most economically feasible and technically viable combination of technologies that satisfy these demands in each market and for each modeling period. Corresponding air pollutant and greenhouse gas emissions are also estimated. A recent version of GCAM (v5.1) has been evaluated by comparing to historical data and to future scenario simulations from other models (Calvin *et al* 2019). GCAM has been widely used to explore global energy and emission scenarios, including the Intergovernmental Panel on Climate Change Special Report on Emissions Scenarios (Nakicenovic *et al* 2000), Representative Concentration Pathways (Thomson *et al* 2011), shared socioeconomic pathways (SSP) (Calvin et *al* 2017), and two-degree mitigation scenarios (Fawcett *et al* 2015).

GCAM-USA is an extension of GCAM with representation of US state-level energy supply and demand markets (Zhou *et al* 2014, Iyer *et al* 2017, JGCRI 2019b). State-level population and economic growth assumptions are calibrated to historical levels through 2015. For future modeling years, population projections are derived from the downscaled shared socioeconomic pathways 2 (SSP2) ‘Middle-of-the-Road’ scenario (Jones and O’Neill 2016, Jiang *et al* 2018), being qualitatively consistent with assumptions made in the rest of the model. State-level per capita GDP growth rates (table S2 is available online at stacks.iop.org/ERL/14/124071/mmedia) are harmonized with regional assumptions in the National Energy Modeling System model used in the 2016 Annual Energy Outlook (AEO) (EIA 2019a).

In contrast to stylized business-as-usual scenarios, this study reflects major air quality and energy policies currently in place, and how these constrain state-level emissions. In previous work (Shi *et al* 2017),we incorporated US air pollutant emission coefficients, pollution controls for coal-fired power plants, and representations of major energy and air quality regulations, and we adjusted the base year emissions and future trends to be consistent with the 2011 National Emission Inventory and its projections (EPA 2015). Here, we adopt improved representations of building sector energy demands, electric vehicle costs, and retirement of existing coal and nuclear electric power plants, and harmonize total electricity generation and electricity generation from coal with the 2018 AEO (EIA 2019b) (SI section 1). Additionally, a nine-state regional cap on electric sector CO_2_ emissions was added to reflect the regional greenhouse gas initiative (RGGI) (RGGI2019).

### State-level PMMC

We project future changes in PMMC based on monetized PM_2.5_-related mortality impacts using estimates of willingness-to-pay to avoid these outcomes. This method has been used by US EPA in benefit-cost analyses of the Clean Air Act (EPA 2011b). Mortality impacts account for over 90% of the total monetized PM_2_._5_ health impact (Martenies *et al* 2015). PM_2.5_, in turn, dominates total air pollutant mortality burdens (Zhang *et al* 2018).

Our initial application of GCAM-USA to model PMMC (Ou *et al* 2018) utilized national average year-, pollutant-, and sector-specific PMMC coefficients (Fann *et al* 2012). Projections of these coefficients to 2050 accounted for national changes in population and economic growth. This paper improves upon Ou *et al* (2018) by applying PMMC coefficients that differ by state, accounting for the spatial heterogeneity of pollutant transport and chemistry, population, and baseline mortality rates. The reduced-complexity model EASIUR (Heo *et al* 2016a), derived from tagged CTM simulations, has estimated all-cause mortality costs of PM_2_._5_ ($/tonne) due to air pollutant emissions (primary PM_2_._5_, SO_2_, NO_*x*_, and NH_3_) from each county in the US in 2005, using the concentration-response function (equation (1)) from the Krewski *et al* (2009) with a relative risk (RR) of 1.06 (95% confidence interval, CI of 1.04–1.08) and a value of statistical life (VSL) of $8.6 million (2010 USD)

(1) $$\Delta \text{PMMC}=y_{0}\cdot \left\{1-\exp\left(-\frac{\ln\text{RR}}{10}\cdot \Delta c\right)\right\}\cdot \text{VSL},$$

where *y*_*0*_, the baseline mortality, is the product of baseline mortality rate and population for given grid, Δ*c* is the change in PM_2_._5_ concentration, and RR is the relative risk, representing the change in mortality rate for an increase of 10 μ*g* m^−3^ in PM_2_._5_ concentration.

In our work, these county-level per-tonne mortality cost estimates are aggregated to the state level for the electricity, industry, transportation, and building sectors in 2005, based on the emission-weighted sum for each sector. These state mortality cost factors reflect the mortality costs across the US of emissions from each state, regardless of where those deaths occur. For some states (e.g. California), most of the health impacts occur in the same state as the emissions. In other states, substantial health impacts may occur in downwind populous states. Growth factors were derived to account for changes in population exposure and baseline mortality rates in future years (Heo *et al* 2016a). Using these growth factors, the 2005 per-tonne mortality cost estimates are adjusted here for each GCAM-USA year (2010–2050 in 5 year increments). VSL also grows over time as a function of GDP per capita, reflecting increased willingness-to-pay (SI section 3). The 95% CI on total PMMC is quantified by applying RR adjustment factors (Heo *et al* 2016a) based on the 95% CI of RR (1.04–1.08) from Krewski et *al* (2009), reflecting the uncertainty in concentration-response function but not other potential sources ofuncertainty (SI section 3).

Besides EASIUR, several other reduced-complexity models have been used to estimate regional PM_2.5_ mortality coefficients, with relatively good agreement for national and sectoral estimates (Gilmore *et al* 2019). Here we use PM_2_._5_ mortality coefficients derived from EASIUR because it fits better with the sectoral-, spatial-, and temporal resolutions of GCAM-USA compared with other publicly available reduced-form approaches (SI section 3).

### Decomposition of changes in PMMC

To quantify the relative importance of various factors to future PMMC changes, a decomposition approach is employed using the following two steps: First, analogous to the Kaya identity (Kaya 1990, Raupach *et al* 2007), the PMMC for a given state and year is expressed as the product of the following driving factors (table 1): population *(D*^*POP*^), GDP per capita (*D*^GDP^), sectoral energy intensity $\left(D_{s}^{ENERGY}\right)$, fuel share $\left(D_{f,s}^{FUEL}\right)$ and PMMC intensity $\left(D_{f,s}^{\text{PM}}\right)$ (equation (2)). Energy intensity (EJ/$) is the energy required to produce one unit of GDP, a measure of the energy efficiency of an economy. Fuel share (ratio) indicates the share of polluting fuels (coal, gas, biomass, and liquids) in each sector. PMMC intensity ($/tonne) represents the PMMC per unit energy use for a technology, as the product of emission intensity (tonne/EJ) and per-tonne mortality costs ($/tonne).

(2) $$M=\sum_{f}\sum_{s}M_{f,s}=\sum_{f}\sum_{s}P\times \frac{G}{P}\times \frac{E_{s}}{G}\times \frac{E_{f,s}}{E_{s}}\;\;\times \frac{M_{f,s}}{E_{f,s}}=\sum_{f}\sum_{s}D^{POP}\times D^{\text{GDP}}\times D_{s}^{ENERGY}\;\;\times D_{f,s}^{FUEL}\times D_{f,s}^{\text{PM}}.$$

Next, the logarithmic mean divisia index (LMDI) approach is adopted to separate the impacts of different factors on the overall changes in PMMC (equation (3)). LMDI has been widely employed for analysis of driving forces and to provide policy-relevant insights, including wind energy supply in China (Lu *et al* 2016) and emission trends of CO_2_ in China (Guan *et al* 2018) and SO_2_ in the US (Lu *et al* 2012). LMDI has been shown to have advantages over other decomposition methods because of its path independence, consistency in aggregation, and ability to handle zero values (Ang 2005, Ang and Wang 2015).

The changes in PMMC for a given region from year *t*_0_ to year *t*_1_ *(*Δ*M)* can be expressed as the sum of contributions from individual factors:

(3) $$\Delta M=\sum_{k}\Delta M_{k}=\sum_{k}\sum_{f}\sum_{s}L\left(w_{f,s}^{t_{0}},w_{f,s}^{t_{1}}\right)\ln\left(\frac{D^{k,t_{0}}}{D^{k,t_{1}}}\right),$$

where *k = POP,* GDP, *ENERGY, FUEL,* PM and $L\left(w_{f,s}^{t_{0}},w_{f,s}^{t_{1}}\right)=\left(M_{f,s}^{t_{0}}-M_{f,s}^{t_{1}}\right)/(\ln\left(M_{f,s}^{t_{0}}\right).\;\ln\left(M_{f,s}^{t_{1}}\right))$ is the logarithmic mean of the mortality costs of fuel f in sector s at times *t*_0_ and *t*_1_

Note that LMDI is fundamentally different from linear regression. For example, in LMDI, the change in the quantity being decomposed is always a linear combination of all contributors, with the residuals being allocated to contributions from individual factors (Lu *et al* 2012, Ang and Liu 2007). In contrast, linear regression typically produces a residual term that represents both model misspecification and confounding factors. Furthermore, LMDI estimates the total effect of each contributor on the PMMC changes, while regression coefficients represent the marginal effect of each contributor at their average level.

## Results

### National air pollutant emissions and PMMC

**Figure 1.**
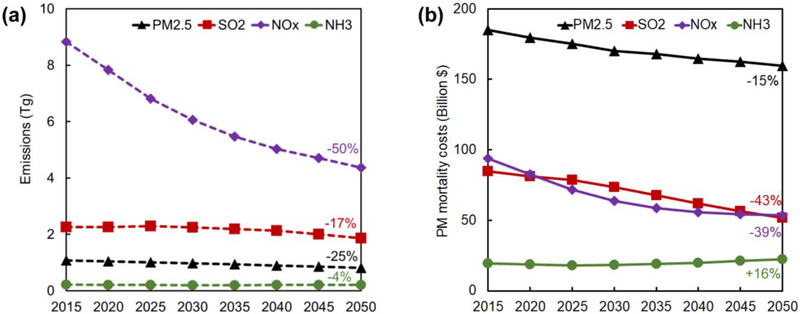
Change in (a) national anthropogenic pollutant emissions (Tg) and (b) PM_2.5_-attributable mortalitycosts (2018$) byemitted pollutant species.

Between 2015 and 2050, emissions of primary PM_2_._5_, NO_*x*_, and SO_2_ are projected to decrease by 25%, 50%, and 17%, respectively (figure 1(a)). Sector-specific pollutant emissions projections are shown in figure S2. The corresponding mortality costs from emissions of primary PM_2_._5_, NO_*x*_, and SO_2_ decrease by 15%, 39% and 43%, respectively (figure 1(b)). The discrepancy between emission and mortality cost changes is a result of pollutant and source category-specific mortality intensity trends (Heo *et al* 2016a), which are simultaneously affected by decreasing baseline mortality rates, increasing population exposure, and increasing VSL in the future. For all modeling years, primary PM_2_._5_ emissions are the greatest source of PMMC. The combined damages from NO_*x*_ and SO_2_ are roughly equivalent to those of primary PM_2_._5_ in 2015, but by 2050 play a lesser role.

**Figure 2.**
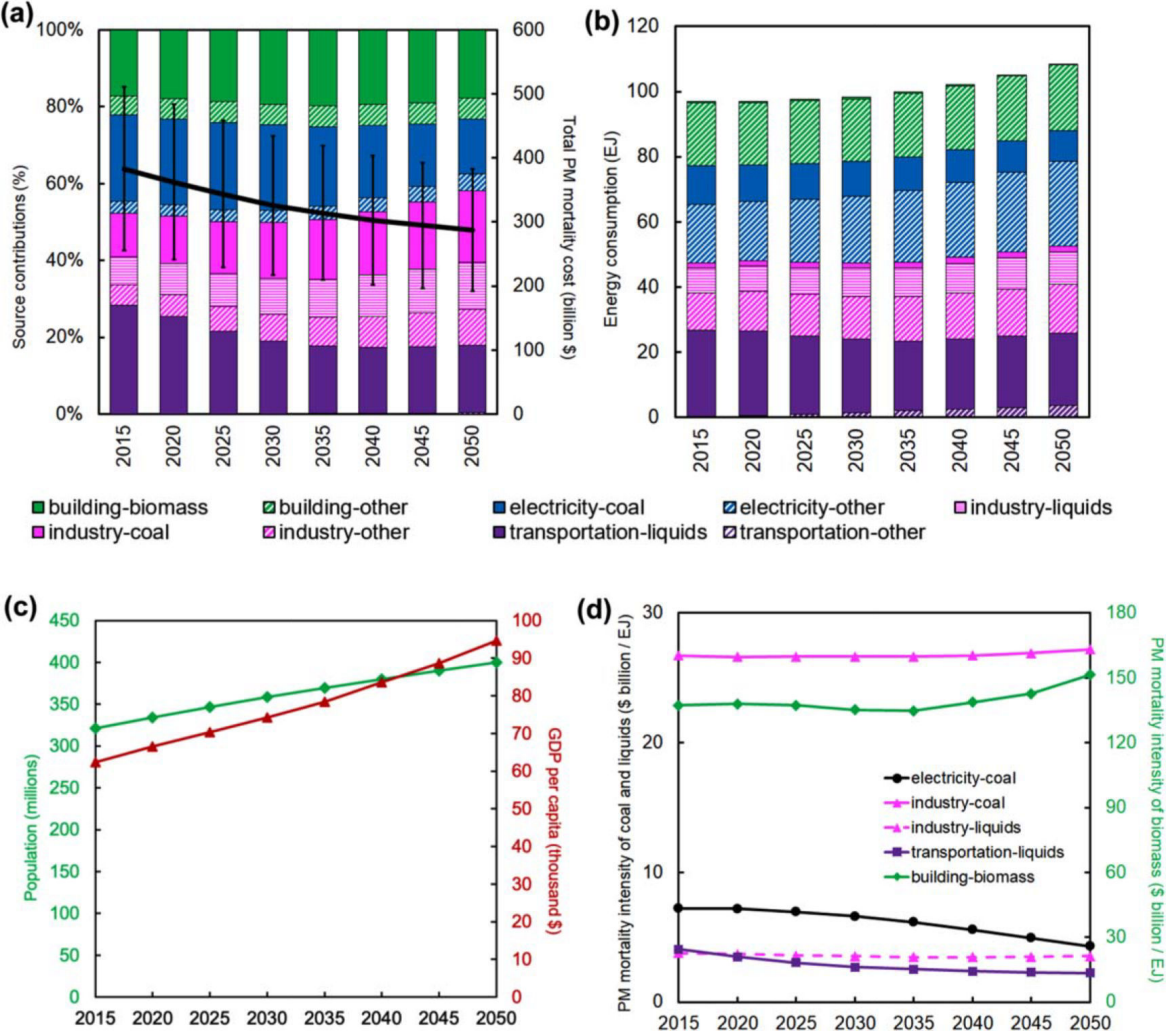
Change in PMMC ($2018) and related indicators in the US from 2015 to 2050. (a) Total PMMC (solid line-right axis) and relative contribution of specific emission source sectors and fuels (bars-left axis); (b) energy consumption by emission sector and fuel combinations; (c) population and GDP per capita ($2018); (d) PM_2.5_ mortality intensity of major emission sources. The 95% CI of total PMMC is calculated by considering the 95% CI of the RR from Krewski *et al* (2009). The shares of major sources (with shares exceeding 10% in anymodeling year) are shown separately, and the categories of ‘-other’ represent aggregated minor contributors to PMMC.

Driven by existing regulations and projected demographic, economic, and technological changes, the national total PMMC (figure 2(a)) decreases 25% from $380 billion in 2015 to $284 billion in 2050. Overall, coal combustion sources maintain a relatively constant share of around 33% of PMMC for all modeling years, with major contributions transitioning from electric sector coal to industrial coal. The contribution of liquid fuels to PMMC drops from 35% in 2015 to 29% in 2050, dominated by the decreasing share attributed to transportation liquids.

Among the expected drivers of PM_2_._5_ mortality trends, energy consumption increases by 12% in 2050 (figure 2(b)), but at a much slower rate than the growth in population (24%) and GDP per capita (52%) (figure 2(c)). The specific source sector-fuel combinations of industrial coal and building sector biomass are major contributors to PMMC (figure 2(a)) despite being minor contributors to overall energy consumption (figure 2(b)). Industrial sources often are not subjected to as stringent emission limits as is the electric sector. Thus, a quantity of coal combusted in the industrial sector typically produces more PM_2_._5_ and precursor emissions than if combusted in the electric sector. Building sector biomass largely consists of wood fireplaces and furnaces, which have high primary PM_2_._5_ emission intensities (figure 2(d)).

Most of the energy consumption in the building and industry sectors comes from gas and electricity. Over the modeling period, coal consumption for electricity production decreases from 12 EJ to 9 EJ (−25%), while the total energy consumption in the electricity sector increases from 30 EJ to 35 EJ (+17%). Together, these trends reflect that more electricity is being produced by clean (i.e. low or zero pollutant emitting) technologies in the future. Figure 2(b) also indicates that liquids remain the dominant fuel in the transportation sector for all modeling years, though liquids’ share of total transportation energy consumption decreases from 99% in 2015 to 86% in 2050, replaced by electric and hydrogen vehicles.

The representation of air pollution regulations in GCAM-USA reduces PM_2.5_ mortality intensity (mortality cost per unit energy input) by decreasing future emission factors (figure 2(d)). Due to regulations limiting coal-fired power plant emissions and Tier 3 motor vehicle and fuel emissions limits, PM_2.5_ mortality intensities of electricity coal and transportation liquids decrease by 40% and 36%, respectively, contributing to their decreasing shares observed in figure 2(a). The PM_2_._5_ mortality intensity of building biomass decreases slightly before 2035, reflecting a gradual turnover from existing wood furnaces to more efficient, EPA-certified wood furnaces and stoves. Note that the temporal trend of PM_2_._5_ mortality intensity is also affected by the increasing VSL and population, as well as by changes in baseline mortality rates (Heo *et al* 2016a). For building sector biomass, the increasing trend of PM_2.5_ mortality intensity after 2035 indicates that the effect of efficiency improvements is overcome by the increase in population exposure.

### LMDI decomposition

**Figure 3.**
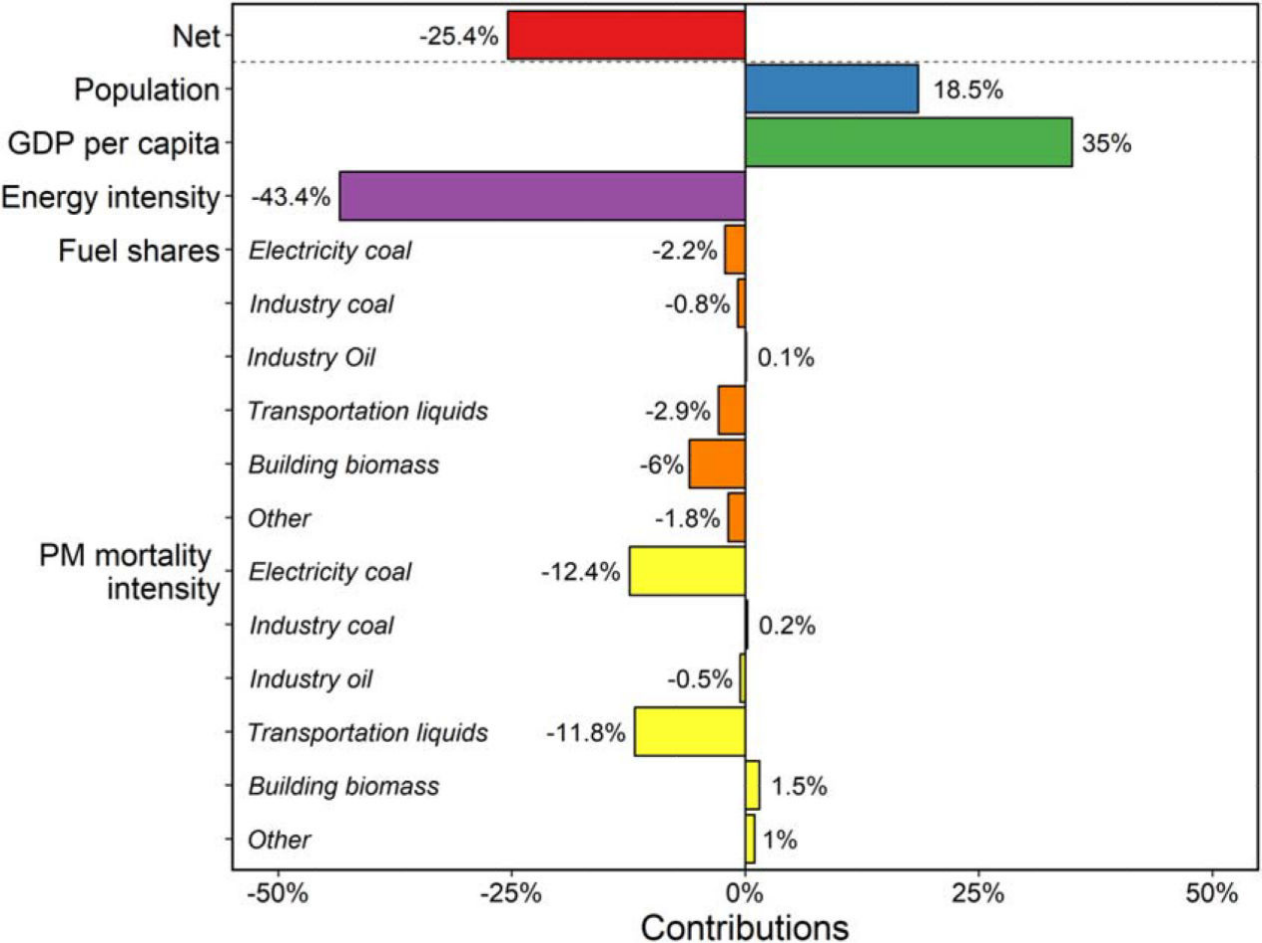
Net change in PMMC and contributions due to changes in population, GDP per capita, energyintensity, fuel shares and PM_2.5_ mortalityintensityfor the contiguous USin2050 relative to 2015.

For the 25% national decrease in the central estimation of PMMC (figure 2(a)), LMDI decomposition reveals that the largest contributor is decreased energy intensity, followed by reduced PM_2 5_ mortality intensities in electric sector coal and transportation liquids (figure 3). In the absence of other factors, these three factors would cause mortality costs to decrease by 68%. These beneficial contributions offset the increases in population and per capita GDP, which in isolation would increase PMMC by 54%.

For electricity coal and transportation liquids, contributions from their decreased PMMC intensity is much greater than from their decreasing fuel shares. The trend of electricity coal is mainly due to CSAPR, which limits electric sector NO_*x*_ and SO_2_ in the eastern US and the New Source Performance Standards (NSPS), which requires all new coal plants to apply controls on NO_*x*_, SO_2_, and PM_2 5_ emissions. Within GCAM-USA, CSAPR is represented by state-specific, electric sector emission constraints, while NSPS is implemented with systematically lower emission factors applied in future modeling years. In the transportation sector, Tier 3 engine and fuel standards are reflected in the model as lower pollutant emissions per unit of fuel consumption over time. Independent of other factors, decreases in the PM_2.5_ mortality intensities of electricity coal and transportation liquids each result in mortality cost decreases of approximately 12%. Changes in building sector biomass produce a net reduction in PMMC, resulting from a decrease in the share of building biomass, contributing −6%, and a small increase in its PM_2 5_ mortality intensity.

Separate LMDI decompositions are further conducted to attribute changes in PMMC to changes in emissions of primary PM_2.5,_ NO_*x*_, and SO_2_ (figures S3–S5). As with the combined results for changes in emissions of all three pollutants (figure 3), decreases in species-specific mortality costs are predominantly due to decreases in energy intensity and are partially offset by increases in population and per capita GDP. However, the second most important drivers of decreasing mortality costs vary by species: building biomass fuel share (causing species-specific mortality costs to decrease by 11.8%, independent of other factors) for primary PM_2 5_, PM_2 5_ mortality intensity of transportation liquids (32.6% decrease) for NO_x_, and PM_2 5_ mortality intensity of electricity coal (45% decrease) for SO_2_. These differences in main contributors further highlight the importance of tailoring air quality management strategies to address specific sectors and pollutants.

### State-level PMMC

**Figure 4.**
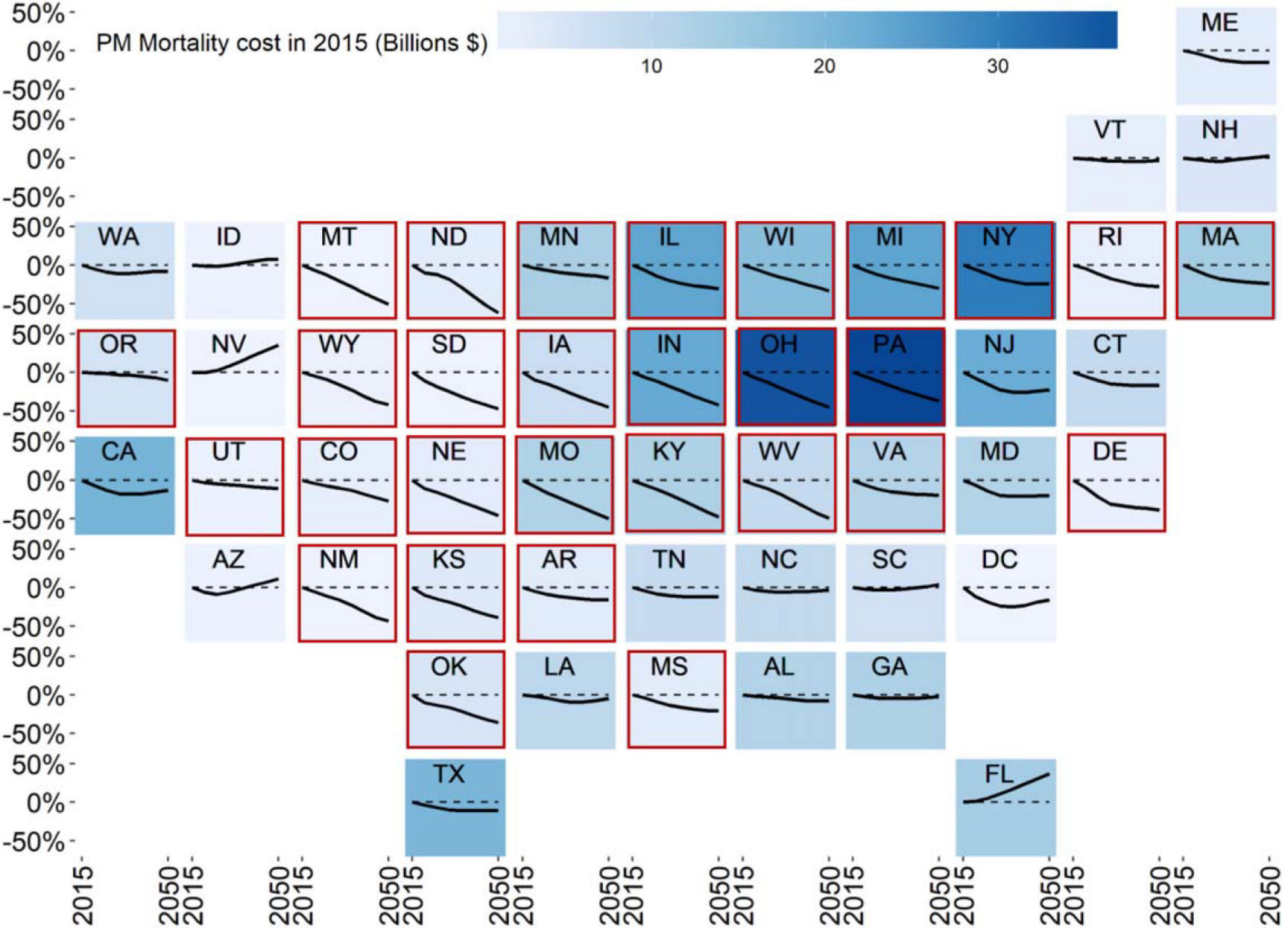
Estimates of PMMC (billion 2018$) in 2015 (colors) and from 2015 to 2050 for the contiguous US states. Panels are organized to suggest geographic locations of states. States in a red box indicate ‘Steadily-decreasing’ states and the rest are ‘Upturn’ states. Full names of US states are shown in table S6.

In addition to decomposing changes in national PMMC, characterizing changes in state-level PMMC and the underlying drivers may be even more important for state and regional energy planning and emission control strategy development. PMMC changes in 2050 vary significantly by state, ranging from −62% (ND) to +37% (FL) (figure 4).

For most states, economic development, as measured by per-capita GDP growth, is the leading contributor to increased PMMC, while decreased energy intensity plays the dominant role in lowering PMMC (table 2). The second largest factor lowering PMMC for most states is the decreased PM_2_._5_ mortality intensity of either electricity coal or transportation liquids. Twelve of the 16 states whose top two contributors are related to electricity coal are CSAPR states.

Besides these general patterns, some states have specific challenges in reducing PMMC. For example, CT, ME, RI, and VT have high heating demand coupled with significant historical use of biomass. Consequently, increases in the share of biomass in the building sector in these states is the second largest contributor to their increased PMMC.

**Figure 5.**
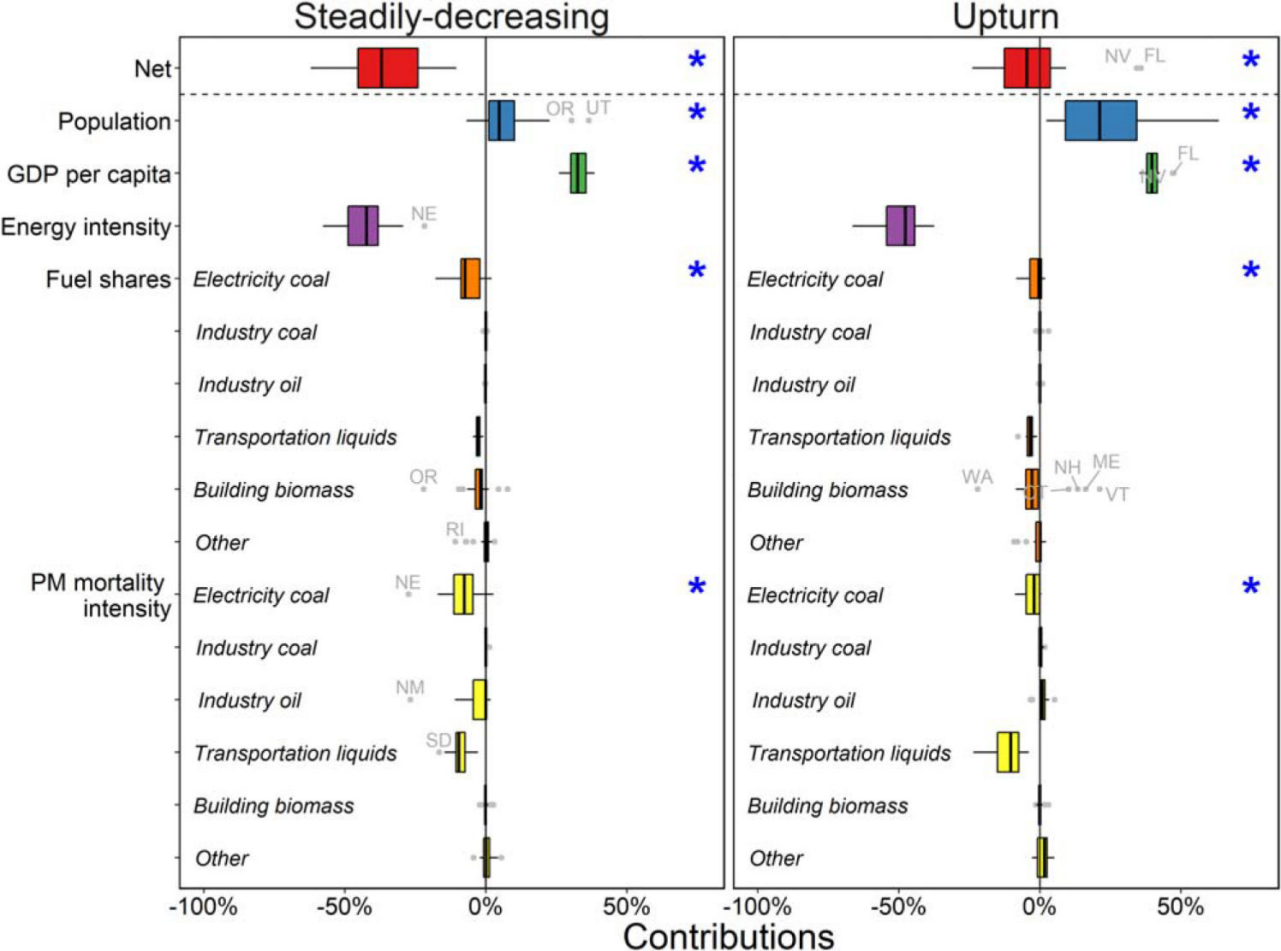
State-specific changes in overall PMMC and contributions due to changes in population, GDP per capita, energy intensity, fuel shares, and PM_2.5_ mortality intensity in 2050 relative to 2015 summarized by states with (left) steadily-decreasing and (right) upturn trends of PMMC. Blue asterisks indicate a significant difference between the two groups (t-test, *p* < 0.001). Labeled states indicate outliers with absolute contributions greater than 10%.

The 48 contiguous US states are categorized into two groups based on the shape of their projected PMMC trends (figure 5). The ‘Steadily-decreasing’ group includes 29 states, with monotonically decreasing PMMC, mostly located in the central US. Of these, Ohio and Pennsylvania have the highest PMMC in 2015. The ‘Upturn’ group includes the remaining 19 states, which have an increasing trend of PMMC either over the entire 2015–2050 period (such as Florida) or starting from an intermediate year (such as Arizona). From a policymaker’s perspective, the Upturn states may indicate state-specific challenges to reducing PMMC in the future, which should be anticipated in the development of long-term air quality management strategies.

In comparing the two groups, the Steadily decreasing states have significantly *(p* < 0.001) greater decreases in state-level PMMC, smaller increases associated with population and per capita GDP growth, and greater decreases in electric sector coal share and PM_2.5_ mortality intensity in 2050. This comparison highlights that population and economic growth can be dominant factors contributing to increased PMMC, especially for the Upturn states. Changes in electricity coal shares and PM_2.5_ mortality intensity have significantly smaller effects among the Upturn states, because these states tend to have smaller shares of electricity coal in 2015. In addition, 8 of these 19 states are within the CSAPR region and their coal fleet emissions largely have been controlled by 2015, leaving less benefit available from turnover to cleaner coal capacity. Contributions from PMMC intensity of transportation liquids are similar among the two groups of states since Tier 3 tailpipe and fuel standards are national policies. Among states with little benefits from decreasing coal use, such as Florida, decreases in the PMMC intensity of transportation liquids play a major role in partially offsetting the effects of population and economic growth.

## Discussion

To our knowledge, this study provides the first systematic quantification of the contributions of changes in population, economic growth, energy intensity, fuel shares, and PM_2_._5_ mortality per unit fuel consumption to projected changes in national and state-level PM_2_._5_ mortality costs. Our results highlight the importance of both socioeconomic and energy system factors in long-term changes in PM_2.5_ mortality.

We find that decreased energy intensity is the largest contributor reducing future PMMC. This is consistent with findings from empirical studies (Levinson 2009, 2015) and past industrial practices (Lovins 2018) that energy efficiency and technology innovation in manufacturing processes can decouple future emissions from population and economic growth.

State-level decompositions elucidate the effect of existing regulatory policies. Federal regulations such as Tier 3 fuel and vehicle standards and pollution limits for new power generation result in the vehicle and power plant fleets gradually becoming considerably lower emitting than their predecessors. Regional emission caps like CSAPR, on the other hand, can have a more immediate impact when implemented.

Besides the general trends identified in state groups (figure 5), our results also highlight states that stand out from the larger groups. For instance, despite the overall beneficial contribution of decreased building sector biomass shares found among the ‘Upturn’ states, NH, ME, and VT face significant challenges due to the increasing share of biomass in the building sector. Also, ME has a much lower contribution from energy intensity improvements and a much higher contribution from decreased PM_2 5_ mortality intensity in electricity coal. This interesting combination may suggest interactions between changes in that state’s overall energy structure and in its power generation.

Several caveats should be mentioned. First, this paper focuses only on one scenario, representing a plausible projection of the evolution of the US energy system and related emissions through 2050, considering existing federal and state emission regulations. We do not attempt to explore alternative scenarios, which could include new regulations, differing technology cost trajectories, or varying socioeconomic conditions. State-level drivers of PMMC could be different under alternative scenarios. Second, the PMMC estimation in this paper relies on EASIUR (Heo *et al* 2016a), thus sharing its major uncertainties in air quality modeling, concentration-response relations, and VSLs (Heo *et al* 2016a, 2016b). For example, EASIUR treats primary organic PM_2.5_ as inert, and included in primary PM_2.5_ emission, and neglects secondary organic PM_25_ (SOA) (Heo et al 2016b) as the understanding and representation of SOA formation is still rapidly evolving in CTMs. SOA can account for half of ambient PM_2 5_ (Jimenez *et al* 2009), depending on season and location (Kim *et al* 2015). Although both biogenic and anthropogenic emissions contribute to SOA formation, anthropogenic emissions generally increase SOA through both volatile organic compounds (VOC) emissions and by enhancing the production of SOA from biogenic sources (Fine *et al* 2008, Riva et *al* 2019). Therefore, the present analysis most likely underestimates the total mortality cost of PM_2.5_ pollution, and omits the effects of VOC emissions and effects on SOA from other precursors such as NO_*x*_. Third, despite additional adjustment for future population, VSL, and baseline mortality rate, the PMMC coefficients are derived from emissions and meteorology in 2005 (Heo *et al* 2016a). While estimates of PM_2 5_ mortality coefficients are considered valid within a factor of two, even over fairly large changes in baseline emissions (Heo *et al* 2016b), it would be preferable to use PM_2_._5_ mortality coefficients determined specifically for future-year emissions and demographics, considering also potential changes in climate and consequent changes in PM_2.5_ (Fiore *et al* 2015). Fourth, EASIUR neglects mortality effects of other air pollutants such as ozone, as well as morbidity effects of PM_2.5_. For PM_2.5_ mortality, EASIUR assumes a log-linear exposure-response function and equal toxicity of all PM_2.5_, while there may be nonlinearities at low levels of exposure and particles from different sources may have different toxicities (Thurston *et al* 2016, Burnett *et al* 2018). Although Lelieveld et al (2015) concluded that the relative source contribution to PM_2.5_ mortality can be very different when using alternative particle toxicity assumptions, the differential toxicities and health effects of specific PM components are still inconclusive (EPA 2009, Cohen et *al* 2017). A systematic quantification of these uncertainties embedded in the CTMs used to develop EASIUR is beyond the scope of the current study. Finally, while we use state-level PMMC coefficients, real-world pollutant exposures occur at a much finer scale, and state-level modeling cannot capture sub-state heterogeneity (Goodkind *et al* 2019). Our state level analysis identifies a number of states facing greater challenges in reducing their PMMC, serving as an initial scoping process. Subsequent analysis with more regional details on both physical and energy emissions modeling would be needed to further identify unique challenges faced by individual sub-state areas and cities (Gulia *et al* 2015, Shan *et al* 2018). In future work, GCAM-USA should be updated to reflect current federal and state-level policies and emerging market trends for new technologies that can significantly reshape energy portfolios.

## Conclusion

Our results suggest that under current policies projected national PM_2.5_ mortality costs will decrease by 25% in 2050 relative to 2015, primarily driven by decreases in energy intensity. Conversely, population and economic growth pose great challenges for mitigating future PMMC in terms of increased exposure, energy demand, and increased valuation of health impacts. Decreases in PMMC per unit consumption of coal in the electric sector and liquids in the transportation sector are the next largest contributors to decreased PMMC. Existing air quality regulations restrain future SO_2_ emissions from electricity coal and NO_x_ emissions from transportation liquids, reducing their corresponding PMMC.

PMMC changes in 2050 vary widely by state, ranging from −62% (ND) to +37% (FL). Among the 48 continental states, 29 states have steadily decreasing PMMC from 2015 to 2050, with relatively small increases in population and per capita GDP, and great decreases in electric sector coal share and PM_2.5_ mortality intensity in 2050. On the other hand, the remaining 19 states with increases in PMMC during 2015–2050 face unique challenges in simultaneously decreasing PMMC and meeting increased energy demands. These states have historically relied on technologies and fuels with high emission intensities, and they also have greater projected population and economic growth. Anticipating this dynamic among these states could help regulators avoid unintended consequences of their policy structures.

## Supplementary Material

Supplement1

## Figures and Tables

**Table 1.** Variables in LMDI decomposition.

Variable	Definition
*f*	Fuels that directly contribute to PM_2.5_ pollution, including coal, gas, refined liquids and biomass
*s*	Energy sectors, including electricity, industry, transportation, and building
*M*	Total PMMC for a given state and year
*M_f, s_*	PMMC attributed to fuel *f* in sector *s* for the state
*P*	State population (table S3)
*G*	State GDP (table S4)
*E_s_*	Total state energy consumption in sector *s*
*E_f, s_*	State energy consumption of fuel *f* in sector *s*
*D^POP^*	State population, same as *P*
*D*^GDP^	State GDP per capita
$D_{s}^{ENERGY}$	State energy intensity in sector *s*
$D_{f,s}^{FUEL}$	Share of fuel *f* in sector *s* for the state
$D_{f,s}^{\text{PM}}$	PM_2.5_ mortality intensity of fuel *f* in sector *s* for the state

**Table 2.** Leading contributors to changes in state PMMC.

State	Leading positive contributors		Leading negative contributors	
	1st	2nd	1st	2nd
AL^a^	GDP	POP	ENERGY	PM_ELEC_COAL
AR	GDP	POP	ENERGY	PM_TRANS_LIQ
AZ	POP	GDP	ENERGY	PM_TRANS_LIQ
CA	GDP	POP	ENERGY	PM_TRANS_LIQ
CO	GDP	POP	ENERGY	PM_TRANS_LIQ
CT	GDP	BUILD_BIO	ENERGY	OTHER
DE	GDP	POP	ENERGY	PM_TRANS_LIQ
FL	POP	GDP	ENERGY	PM_TRANS_LIQ
GA^a^	GDP	POP	ENERGY	PM_TRANS_LIQ
IA^a^	GDP	PM_OTHER	ENERGY	PM_ELEC_COAL
ID	GDP	POP	ENERGY	PM_TRANS_LIQ
IL^a^	GDP	POP	ENERGY	PM_ELEC_COAL
IN^a^	GDP	POP	ENERGY	PM_ELEC_COAL
KS^a^	GDP	POP	ENERGY	PM_ELEC_COAL
KY^a^	GDP	POP	ENERGY	PM_ELEC_COAL
LA	GDP	POP	ENERGY	PM_TRANS_LIQ
MA	GDP	POP	ENERGY	PM_TRANS_LIQ
MD^a^	GDP	POP	ENERGY	PM_TRANS_LIQ
ME	GDP	BUILD_BIO	ENERGY	PM_TRANS_LIQ
MI^a^	GDP	OTHER	ENERGY	PM_ELEC_COAL
MN^a^	GDP	POP	ENERGY	PM_TRANS_LIQ
MO^a^	GDP	POP	ENERGY	PM_TRANS_LIQ
MS	GDP	POP	ENERGY	PM_TRANS_LIQ
MT	GDP	POP	ENERGY	ELEC_COAL
NC^a^	GDP	POP	ENERGY	PM_TRANS_LIQ
ND	GDP	OTHER	ENERGY	PM_ELEC_COAL
NE^a^	GDP	OTHER	PM_ELEC_COAL	ENERGY
NH	GDP	POP	ENERGY	OTHER
NJ^a^	GDP	POP	ENERGY	PM_TRANS_LIQ
NM	GDP	PM_ELEC_COAL	ENERGY	PM_INDUS_LIQ
NV	POP	GDP	ENERGY	PM_TRANS_LIQ
NY^a^	GDP	PM_OTHER	ENERGY	PM_TRANS_LIQ
OH^a^	GDP	OTHER	ENERGY	PM_ELEC_COAL
OK	GDP	POP	ENERGY	PM_ELEC_COAL
OR	GDP	POP	ENERGY	BUILD_BIO
PA^a^	GDP	POP	ENERGY	ELEC_COAL
RI	GDP	BUILD_BIO	ENERGY	OTHER
SC^a^	GDP	POP	ENERGY	PM_TRANS_LIQ
SD	GDP	PM_OTHER	ENERGY	PM_TRANS_LIQ
TN^a^	GDP	POP	ENERGY	PM_TRANS_LIQ
TX^a^	GDP	POP	ENERGY	PM_TRANS_LIQ
UT	GDP	POP	ENERGY	ELEC_COAL
VA^a^	GDP	POP	ENERGY	PM_TRANS_LIQ
VT	GDP	BUILD_BIO	ENERGY	PM_TRANS_LIQ
WA	GDP	POP	ENERGY	BUILD_BIO
WI^a^	GDP	POP	ENERGY	PM_TRANS_LIQ
WV^a^	GDP	OTHER	ENERGY	ELEC_COAL
WY^a^	GDP	PM_INDUS_COAL	ENERGY	ELEC_COAL

Note. GDP: GDP per capita

POP: population

ENERGY: energy intensity

ELEC_COAL: share of coal in electric sector

BUILD_BIO: share of biomass in building sector

OTHER: share of ‘other’ fuels

PM_ELEC_COAL: PM_2.5_ mortality intensity of electricity coal

PM_INDUS_COAL: PM_2.5_ mortality intensity of industry coal

PM_TRANS_LIQUIDS: PM_2.5_ mortality intensity of transportation liquids

PM_INDUS_LIQUIDS: PM_2.5_ mortality intensity of industry liquids

PM_OTHER: PM_2.5_ mortality intensity of ‘other’ fuels.

a States regulated by the cross state air pollution rule.
