# Supplementary material for: State-level drivers of future fine particulate matter mortality in the United States
Source: Environ Res Lett. Author manuscript; Available in PMC 2020 Dec 18. (PMC7055525; doi:10.1088/1748-9326/ab59cb)
Supplement: Supplement1 [file NIHMS1548194-supplement-Supplement1.pdf]

Supporting Information

State-level drivers of future fine particulate matter mortality in the United States

Yang Ou[1,2,3], Steven J. Smith[4], J. Jason West[3], Christopher G. Nolte[2], and Daniel H. Loughlin[2,*]

[1] Oak Ridge Institute for Science and Education

[2] Center for Environmental Measurement and Modeling, U.S. Environmental Protection Agency, RTP, NC

[3] Environmental Sciences & Engineering, University of North Carolina at Chapel Hill

[4] Joint Global Change Research Institute, Pacific Northwest National Laboratory, College Park, MD

* Corresponding author. Tel.: +1 919 541 3928; Fax: +1 919 541 7885;

Email address: Loughlin.Dan@epa.gov

The following supporting information provides text, tables, and figures pertaining to 1) updated GCAM-USA policy reference scenario; 2) GDP and population assumptions in GCAM-USA; 3) State-specific $PM_{2.5}$ health impact coefficients; 4) LMDI results by pollutant.

six supporting tables and five supporting figures

Section S1: Updated GCAM-USA policy reference scenario

The current paper made significant effort in improving GCAM-USA's representation of several key energy parameters, covering all major sectors. These modifications are guided by recent market trends and major technical reports.

As discussed in the main text, regular updates of GCAM-USA to include more recent market trends and updated federal and state-level policies allow the model to provide better insights for integrated energy and air quality planning at the state level.

Table S1. Energy-related modifications in GCAM-USA

**Sources**	**Reference**
Electricity	
Harmonization of total electricity generation to Annual Energy Outlook (AEO) 2018	EIA, 2018
Harmonization of electricity generation from coal to AEO 2018	EIA, 2018
Updated wind and solar technology cost to reflect more recent market trends	Iyer et al. 2017
Harmonization of electricity generation from wind and solar in 2015 and 2020 to AEO 2018	EIA, 2018
Updated nuclear power availability assumptions	S&P Global Platts, 2018 (market trend)
Electricity technology specific requirement in California and Vermont (no new coal in California, and no nuclear in Vermont)	California Senate Bill No. 1368, 2006; Vermont DPS, 2016
Industry	
No coal-to-liquids and gas-to-liquids in refineries	Market trend
Buildings	
Harmonization of total building energy use to AEO 2018	EIA, 2018
Only LED and CFL bulbs allowed after 2010	H.R.6, 2007
Wood furnace efficiency improvement	EPA, 2015a
Transportation	
Updated battery electricity vehicle costs	UCS, 2017

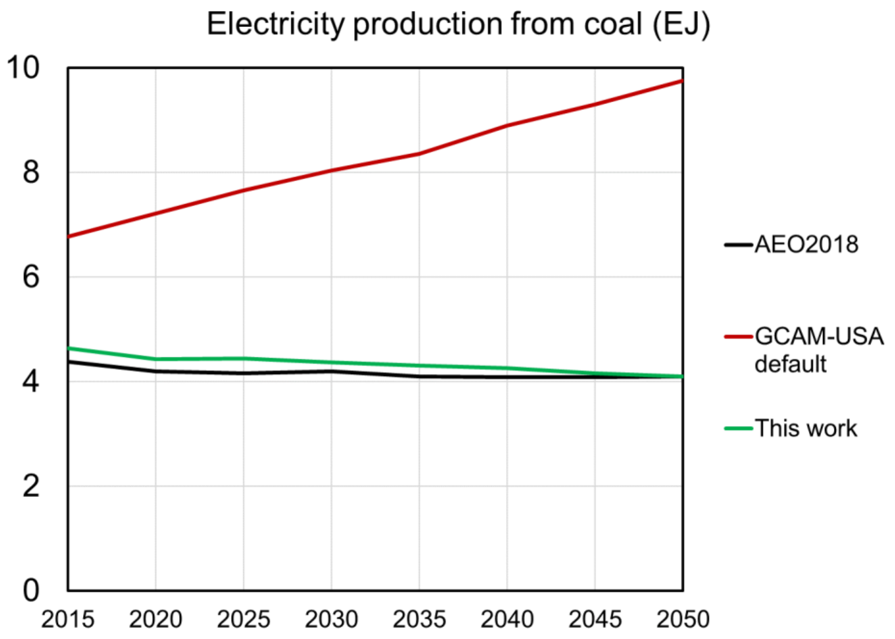

(a)

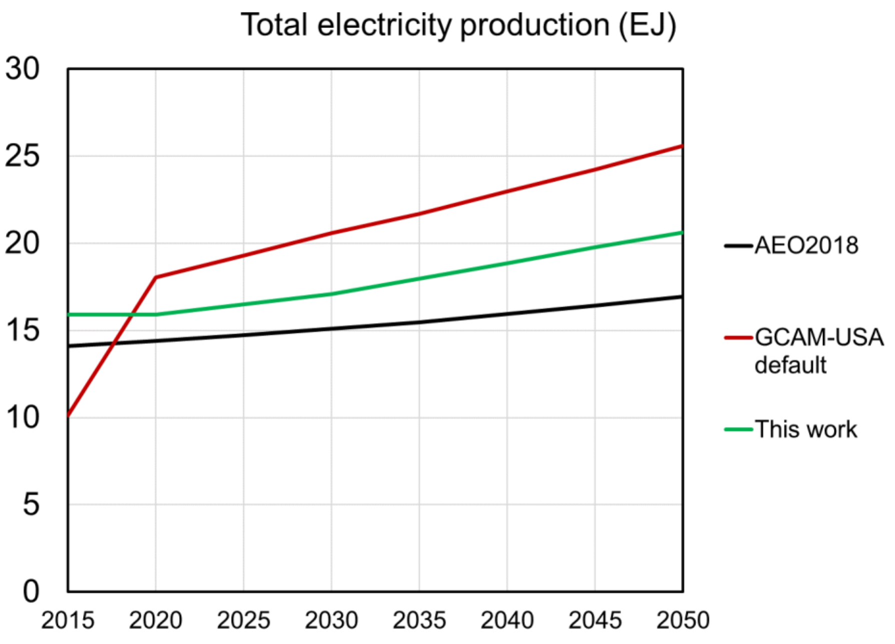

(b)

Figure S1 Electricity production from coal (EJ) (a) and total electricity generation (EJ) (b) in this work, compared with projections from GCAM-USA default (no policy) and 2018 AEO

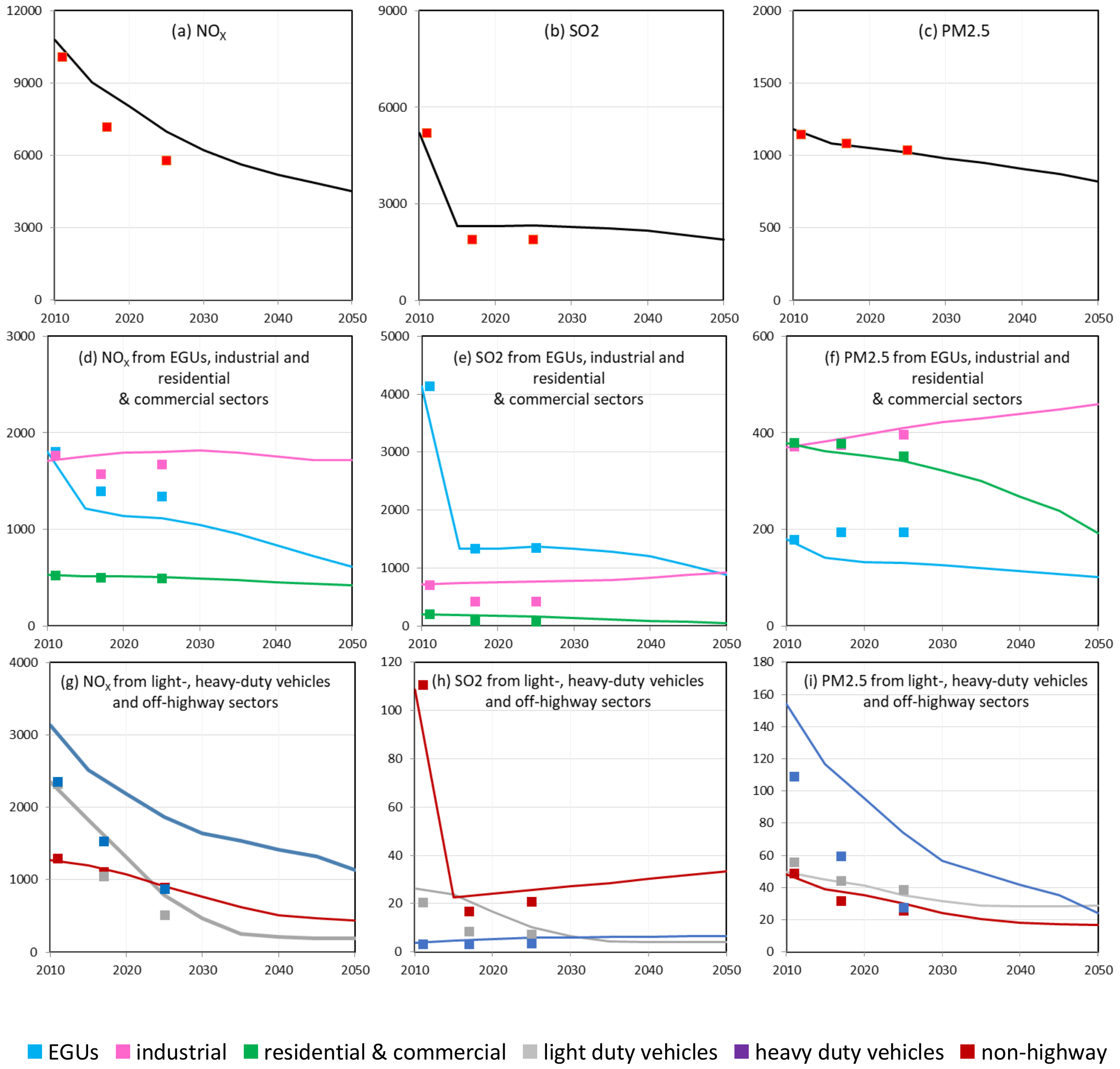

Figure S2 Updated GCAM-USA policy reference scenario emissions projections (lines) compared to EPA 2011 emissions modeling inventory and projections (squares). Units are metric tons.

Section S2: GDP and population assumptions in GCAM-USA

Table S2 Assumption of average GDP growth rates in GCAM-USA and AEO 2018 (EIA, 2018)

	2017-2020	2021-2030	2031-2040	2041-2050	2017-2050
AEO 2018	2.4%	2.0%	2.0%	1.9%	2.0%
	2015-2020	2020-2030	2030-2040	2040-2050	2015-2050
GCAM-USA	2.1%	2.0%	1.9%	2.0%	2.0%

* GCAM-USA uses 5-year time steps, so some comparison periods are not exactly matched

Table S3 State-level population in GCAM-USA (Units: thousand) (JGCRI, 2019)

Region	2015	2020	2025	2030	2035	2040	2045	2050
AL	4,833	4,889	4,947	4,997	5,047	5,106	5,181	5,282
AR	7,210	8,114	9,118	10,192	11,177	12,033	12,735	13,262
AZ	3,004	3,088	3,170	3,242	3,308	3,372	3,439	3,515
CA	39,192	41,120	43,035	44,868	46,500	47,952	49,291	50,578
CO	5,253	5,478	5,714	5,960	6,179	6,374	6,553	6,725
CT	3,619	3,649	3,654	3,631	3,622	3,629	3,658	3,718
DC	939	973	998	1,014	1,030	1,046	1,064	1,087
DE	575	544	514	487	467	455	450	453
FL	20,662	22,749	25,109	27,645	29,946	31,936	33,582	34,858
GA	10,316	10,906	11,470	11,985	12,444	12,850	13,221	13,572
IA	1,681	1,791	1,900	2,009	2,105	2,190	2,264	2,330
ID	12,965	13,069	13,132	13,151	13,195	13,280	13,430	13,668
IL	6,589	6,682	6,757	6,809	6,866	6,936	7,031	7,165
IN	3,053	3,039	3,003	2,948	2,912	2,897	2,906	2,946
KS	2,894	2,925	2,945	2,950	2,961	2,980	3,014	3,068
KY	4,414	4,477	4,529	4,570	4,614	4,664	4,731	4,822
LA	4,584	4,617	4,645	4,659	4,680	4,714	4,770	4,856
MA	1,353	1,368	1,370	1,359	1,354	1,355	1,365	1,387
MD	6,056	6,322	6,560	6,775	6,968	7,144	7,315	7,491
ME	6,635	6,713	6,774	6,808	6,851	6,910	6,998	7,128
MI	9,994	10,060	10,046	9,973	9,937	9,949	10,026	10,186
MN	5,528	5,740	5,925	6,083	6,227	6,361	6,498	6,647
MO	3,000	3,022	3,037	3,043	3,055	3,076	3,111	3,167
MS	6,118	6,233	6,330	6,411	6,490	6,575	6,679	6,813
MT	1,018	1,039	1,051	1,053	1,056	1,063	1,075	1,095
NC	1,842	1,852	1,857	1,854	1,857	1,865	1,884	1,917
ND	3,061	3,446	3,845	4,238	4,596	4,905	5,160	5,358
NE	1,378	1,439	1,492	1,541	1,584	1,624	1,662	1,702
NH	8,993	9,170	9,311	9,420	9,529	9,649	9,798	9,992
NJ	2,120	2,159	2,176	2,157	2,147	2,147	2,163	2,197
NM	19,408	19,388	19,294	19,127	19,037	19,043	19,181	19,482
NV	10,196	10,880	11,596	12,318	12,959	13,517	14,002	14,423
NY	670	663	651	633	620	613	612	619
OH	11,544	11,522	11,450	11,334	11,266	11,260	11,334	11,508
OK	3,818	3,885	3,962	4,036	4,105	4,173	4,249	4,340
OR	4,046	4,284	4,548	4,820	5,062	5,272	5,456	5,617
PA	12,780	12,824	12,800	12,697	12,643	12,651	12,746	12,947
RI	1,070	1,081	1,081	1,070	1,064	1,064	1,071	1,088
SC	4,819	4,993	5,150	5,286	5,409	5,524	5,642	5,770

SD	824	827	824	818	815	816	823	836
TN	6,604	6,869	7,144	7,414	7,655	7,872	8,077	8,280
TX	27,121	29,136	31,310	33,615	35,673	37,454	38,969	40,232
UT	2,964	3,177	3,417	3,672	3,900	4,097	4,264	4,403
VA	643	658	668	672	677	684	693	706
VT	8,444	8,871	9,287	9,691	10,051	10,370	10,663	10,944
WA	7,134	7,608	8,161	8,755	9,285	9,744	10,134	10,461
WI	1,839	1,813	1,773	1,716	1,677	1,654	1,651	1,669
WV	5,819	5,925	5,989	6,018	6,054	6,105	6,182	6,296
WY	571	572	569	559	553	550	552	560

Table S4 State-level GDP in GCAM-USA (Units: Million $1990) (JGCRI, 2019)

Region	2015	2020	2025	2030	2035	2040	2045	2050
AL	124081	133661	143147	152784	162715	175279	188364	205404
AR	69848	76459	83075	89774	96589	104834	113236	123795
AZ	210578	252354	300146	354503	409932	469909	526715	586694
CA	1484560	1658640	1837290	2024050	2211880	2428680	2644040	2901930
CO	175950	195389	215713	237744	259901	285467	310828	341192
CT	163925	176006	186543	195869	206021	219788	234638	255088
DC	55775	56191	56194	56258	56885	59013	61814	66557
DE	43566	48072	52188	56028	60011	64890	69908	76390
FL	609520	714620	834833	971213	1109330	1259670	1402880	1557550
GA	331377	373055	415268	458491	501970	551919	601413	660356
IA	81751	86655	90631	94011	97919	103724	110195	119488
ID	38807	44029	49437	55234	61024	67600	74015	81475
IL	444062	476662	506940	536429	567529	608178	651397	709089
IN	185226	200026	214087	227955	242379	260707	279897	305087
KS	88936	95719	102004	107965	114268	122449	131165	142809
KY	111227	120133	128628	137144	146004	157145	168823	184048
LA	144230	154692	164722	174576	184911	198318	212533	231426
MA	269155	289984	309714	328899	348998	374801	402007	437979
MD	214292	238216	261624	285503	309624	338004	366549	401498
ME	38062	40980	43438	45530	47832	50968	54378	59101
MI	315579	338271	357534	375040	394033	420059	448327	487189
MN	189717	209772	229182	248621	268364	291894	315803	345532
MO	184048	199672	214625	229684	245174	264472	284533	310445
MS	65673	70446	74932	79332	83982	90036	96442	105012
MT	24243	26348	28209	29864	31580	33848	36253	39498
NC	307335	349227	393954	442186	490525	544784	597683	658510
ND	17362	18295	19013	19535	20175	21239	22458	24296
NE	52941	56682	60155	63459	67023	71671	76680	83455
NH	45660	50774	55720	60809	65909	71950	77985	85422
NJ	354925	385388	414174	442756	472266	509185	547605	597322
NM	50608	54882	58545	61321	64361	68529	73120	79439
NV	114246	136959	161744	188374	215410	244782	272726	302904
NY	801245	852343	897762	940402	986939	1051190	1121380	1218270
OH	347726	369577	388723	406580	426145	453503	483461	525054
OK	98952	107220	115733	124573	133601	144610	155946	170374
OR	111868	126132	141727	158711	175755	194902	213625	235238
PA	378646	404597	427432	448008	470392	501174	534779	581026
RI	33819	36383	38509	40276	42231	44966	47936	52087
SC	125549	138521	151224	164009	176963	192430	208156	227697

SD	21139	22593	23826	24992	26256	27991	29900	32486
TN	189783	210203	231390	253737	276250	302479	328698	360416
TX	833492	953504	1084510	1230300	1376710	1539060	1695950	1872800
UT	75657	86355	98304	111624	125010	139829	154130	170233
VA	291845	326493	361771	398892	436236	479231	521894	572934
VT	17961	19572	21031	22355	23747	25547	27413	29871
WA	248413	282104	320287	363062	406005	453671	499714	551746
WI	167234	181327	193993	205973	218487	234598	251596	274072
WV	37286	39143	40516	41435	42698	44839	47403	51256
WY	19239	20523	21608	22431	23398	24779	26338	28580

Section S3 State-specific $PM_{2.5}$ health impact coefficients

Heo et al. (2016a) estimated the avoided premature mortality cost associated with a 1-tonne reduction in primary $PM_{2.5}$ or inorganic $PM_{2.5}$ precursor emissions (SO_2, NO_x and NH_3) at US county-level resolution. A reduced-complexity model (Estimating Air Quality Social Impacts Using Regression, EASIUR) was derived from tagged chemical transport model simulations based on 2005 conditions. Heo et al. (2016a) further conducted a supplementary analysis using 2040 population (in general being consistent with GCAM's population assumption) and baseline mortality rates and derived "growth factors" to allow users to adjust 2005 estimates to future years. Heo et al. (2016b) provided guidance for deriving sector-specific impact coefficients based on emission-weighted averages of county-level impact coefficients.

Besides EASIUR, several other reduced-complexity models have been used to estimate regional $PM_{2.5}$ mortality coefficients, such as InMAP (Tessum et al. 2017) and AP2 (Muller 2014). Their model structures and mechanisms can be very different. For example, AP2 employs a Gaussian dispersion model to represent atmospheric transport. However, Gaussian dispersion models assume that meteorological conditions at the source are held constant for all downwind areas, limiting the model's ability to predict secondary $PM_{2.5}$ formation (Heo et al., 2016). InMAP estimates annual-average changes in primary and secondary $PM_{2.5}$ concentrations attributable to annual changes in precursor emissions, based on pre-processed physical and chemical information from output of a state-of-the-science chemical transport model. The default pre-processed information uses 2005 conditions. While InMAP links changes in $PM_{2.5}$ concentrations to its precursor emissions, it does not provide health impact estimations like EASIUR or AP2. Heo et al. (2016b) provided a systematic comparison and discussion of the difference between EASIUR estimates and other tools for 2005 estimations, and found that current reduced models including EASIUR showed relatively good agreement for aggregated estimates such as national or sectoral averages in 2005, while substantial differences may exist when estimating secondary species and/or with a small spatial domain.

EASIUR is based on a full-scale chemical transport model, the Comprehensive Air Quality Model with extensions (CAMx), and the Particulate Matter Source Apportionment Technology (PSAT) to track the contribution of emissions from certain sources to average $PM_{2.5}$ levels and health impacts (Heo et al., 2016a). This study used $PM_{2.5}$ mortality coefficients derived from

EASIUR because it fits better with the sectoral-, spatial-, and temporal resolutions of GCAM-USA, compared with other publicly available reduced-form approaches. Gaussian dispersion models have some limitations in secondary $PM_{2.5}$ formations, and InMAP does not provide health impact estimations. More importantly, neither InMAP nor AP2 provides long-term projections, while the marginal health impact estimations provided by EASIUR can be adjusted to future years using factors provided by Heo et al. (2016a) and can be aggregated to major energy sectors using the method in Heo et al. (2016b).

Although EASIUR is preferred for the current study among other reduced-complexity modeling approaches, a number of sources of uncertainty should be acknowledged. The Supplementary Information of Heo et al. (2016b) quantified major sources of its uncertainty in the estimated marginal social costs by comparing uncertainties from regional air quality modeling (area covering 500,000 km^2 or larger), sub-regional air quality modeling (area covering less than 500,000 km^2), concentration-response relation, and value of a statistical life (VSL). Uncertainty from air quality modeling is separately considered for each season, based on absolute values of mean fractional biases of CAMx predictions against observations and EASIUR's 95% confidence intervals. Uncertainty from the concentration-response relation is quantified as the 95% confidence intervals of two $PM_{2.5}$ relative risk estimates from Krewski et al. (2009) and Lepeule et al. (2012). Uncertainty from VSL is represented as the 95% confidence intervals of a Weibull distribution fitted to 26 VSL studies (Abt Associates, 2010). In summary, the major uncertainty in EASIUR estimates come from the concentration-response relationship (-33% to +270%), as well as VSL (-90% to +180%). Although it would be feasible to adopt alternative concentration-response relationship (relative risk) and VSL to quantify uncertainties from these dominant factors in estimating PMMC in this work, this would simply scale results uniformly up or down. The LMDI results decomposing changes of PMMC will not be affected since both the concentration-response relationship and VSL are applied uniformly across all spatial domains and energy sectors.

Marginal mortality costs of $PM_{2.5}$ damages in EASIUR are assessed assuming the primary $PM_{2.5}$ is non-volatile and inert, so any evaporation will make actual damages lower. Therefore, for primary $PM_{2.5}$ species that are inert and non-volatile, EASIUR estimates have no *a priori* bias (although still uncertain). For primary $PM_{2.5}$ species that are semi-volatile, particularly primary

organic $PM_{2.5}$, the actual marginal damages are expected to be lower, by an amount that is difficult to quantify. In the context of the current paper, PMMC for primary $PM_{2.5}$, therefore, considers both organic and inorganic $PM_{2.5}$.

EASIUR does not include the formation of secondary organic $PM_{2.5}$ aerosol (SOA). As noted in the main text, omitting SOA underestimates the total mortality cost of $PM_{2.5}$ pollution. Fann et al. (2013) found that the $PM_{2.5}$ health damages attributed to the source category "Secondary organics & biogenic" are comparable with damages of "Industrial point", "EGU", or "Mobile" sources in 2016, although contributions from anthropogenic and biogenic sources are not separated in that work making that likely an overestimate of secondary anthropogenic contributions. Although both biogenic and anthropogenic emissions contribute to SOA formations, anthropogenic emissions generally enhance SOA formation not primarily as precursors but by increasing the production of SOA from biogenic sources (Fine et al. 2008). Since the anthropogenic precursor emissions will decrease in future (Figure S2), contributions from anthropogenic emissions to SOA formation and the associated health impact may decrease. However, because of non-linear reactions in SOA formation and uncertainties in quantifying biogenic emissions, it is still a scientific and analytical challenge to completely quantify the impact of omitting SOA in the current study. We note that SOA will be most important in summer when biogenic emissions and chemical oxidation rates peak (Fine et al. 2008).

This work estimated $PM_{2.5}$ mortality costs from 2015 to 2050 in five-year increments, considering impacts from emission location, population growth, baseline morality changes, and changes in VSL. Starting from county-level estimates for all sectors based on 2005 conditions provided by Heo et al. (2016a), the following two steps were conducted:

Step 1: Obtaining future-year county-level $PM_{2.5}$ mortality cost coefficients

The public version of EASIUR reports county-level per-tonne mortality cost coefficients ($/tonne) by pollutant for 2005. Heo et al. (2016a) additionally derived impact coefficients using 2040 population and baseline mortality rate projections $\left(S_{2040}^{x,y}\right)$. Together with the original 2005 estimates $\left(S_{2005}^{x,y}\right)$, a location (x, y) and pollutant-specific growth factor $F_p^{x,y}$ was derived (Equation S1). These growth factors range from 0.995 to 1.015 for all pollutants, accounting for changes in population and baseline mortality rates at the county level. The relatively small range

of these growth factors is because the increasing trend of population growth is partially offset by the decreasing trend of baseline mortality rate.

$$F_p^{x,y} = \left(\frac{S_{2040}^{x,y}}{S_{2005}^{x,y}}\right)^{2040-2005}$$

(Equation S1)

Using these growth factors, Heo et al. (2016a) suggested that for future year (n), the corresponding $PM_{2.5}$ mortality cost coefficients $\left(S_n^{x,y}\right)$ can be obtained using Equation S2:

$$S_n^{x,y} = S_{2005}^{x,y} \times \left(F_p^{x,y}\right)^{n-2005}$$

(Equation S2)

Using this approach, we obtained county-level $PM_{2.5}$ mortality cost coefficients for future modeling years.

Step 2: Obtaining state-level $PM_{2.5}$ mortality cost coefficients by sector

These county-level all-sector mortality estimates ($M_{C,Y,A}$) were then aggregated into state-level estimates for each sector for all modeling years based on the emission-weighted sum for each sector (Equation S3). Therefore, for any given pollutant (primary $PM_{2.5,}$ $NO_{x,}$ and $SO_{2,}$) from a given sector (electric, industry, transportation, buildings) in a given modeling year (2005 to 2050 with 5-year steps), the state level mortality cost coefficient can be expressed as

$$M_S = \frac{\sum_C M_C * E_C}{\sum_C E_C}$$

(Equation S3)

Where

S = US state

C = county within State S

M = $PM_{2.5}$ mortality cost coefficients ($/tonne)

E = annual total emission (tonne/yr)

Step 3: readjust $PM_{2.5}$ mortality cost coefficients based on GCAM-USA's GDP per capita assumptions

So far the state-level $PM_{2.5}$ mortality cost coefficients by sector in future years only consider future population, baseline mortality rate, and future emissions. To consider the increasing trend of willingness to pay to avoid adverse health impacts, we use the national per capita GDP assumptions in GCAM-USA (Table S5) to adjust VSLs for future years, based on an income elasticity of 0.5 (Viscusi & Aldy, 2003).

When developing "growth factors", Heo et al. (2016a) used 2040 population embedded within BenMAP v.1.1 (EPA, 2015b) to account for the effect of population growth. Although the 2040 population may not be exactly the same as GCAM-USA's 2040 population assumption, one would need to re-calculate the entire EASIUR based on GCAM-USA's population assumption in 2040, or even every future modeling year, in order to fully address the discrepancy in population assumption.

Step 4: adjusting PMMC with alternative relative risks

The central estimations of PMMC in this paper are based on the relative risk (RR) of 1.06 per 10 $\mu g/m^3$ increases in $PM_{2.5}$ concentration (Krewski et al. 2009). To propagate uncertainty in $PM_{2.5}$ mortality coefficients to PMMC related to concentration-response functions, we report the 95% CI of total PMMC in Fig. 2a based on the 95% CI of the RR (1.04-1.08) reported in Krewski et al. (2009), using the relative risk adjustment factor (F_R) method provided by Heo et al. (2016a). F_R is estimated by Equation S4, where R is the alternative relative risk.

$$F_R = \frac{R - 1.0}{1.06 - 1.0}$$

(Equation S4)

Here we apply R = 1.04 and 1.08 in Equation S4 to derive the upper and lower F_R, which are used to scale the central estimation of total PMMC.

Table S5 Projected U.S. per capita income (GDP) and value of a statistical life (VSL) in all modeling years.

Year	Per capita GDP[1]	VSL[2]
2005	59.2	**9.5**
2010	58.1	9.3
2015	62.4	9.7
2020	66.5	10.0
2025	70.3	10.3
2030	74.3	10.6
2035	78.4	10.9
2040	83.6	11.4
2045	88.6	11.7
2050	94.7	12.2

[1] In million 2018 dollars at a Market Exchange Rate basis

[2] In million 2018 dollars; 2005 VSL in bold red is used as benchmark to translate VSLs in future years

Section S4 LMDI results by pollutant

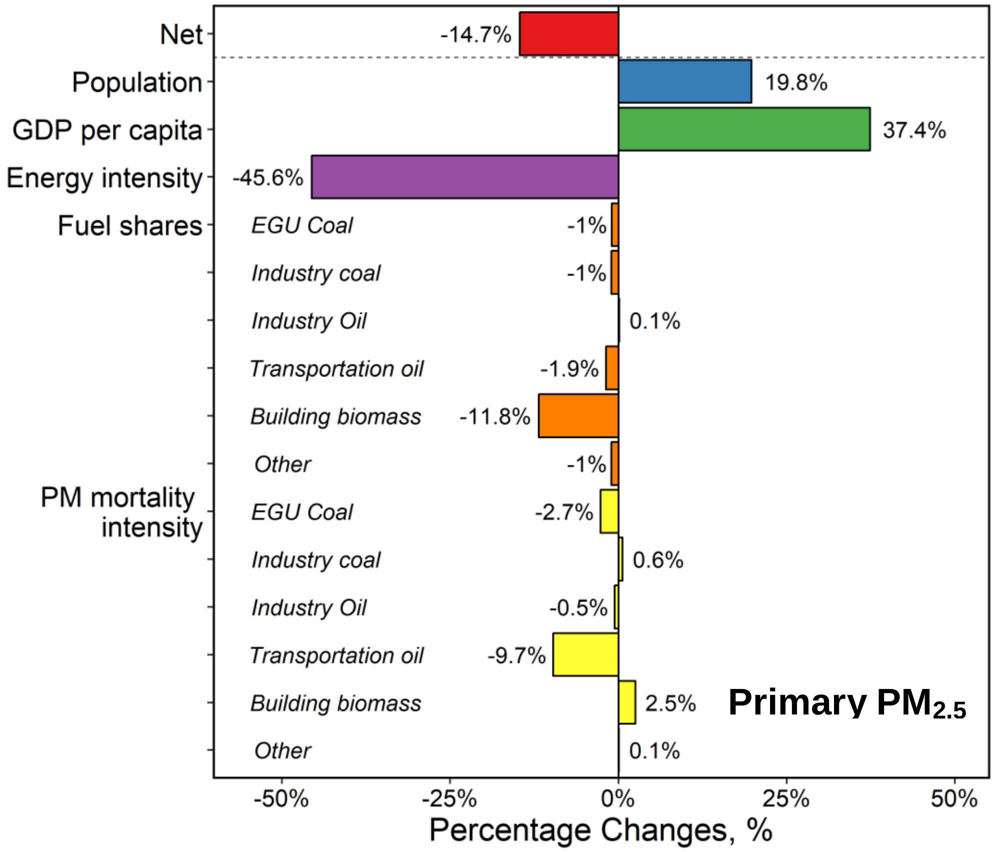

Figure S3 Net change in $PM_{2.5}$ mortality costs attributed to primary $PM_{2.5}$ emissions and contributions due to changes in population, GDP per capita, energy intensity, fuel shares, and $PM_{2.5}$ mortality intensity for the contiguous U.S. in 2050 relative to 2015.

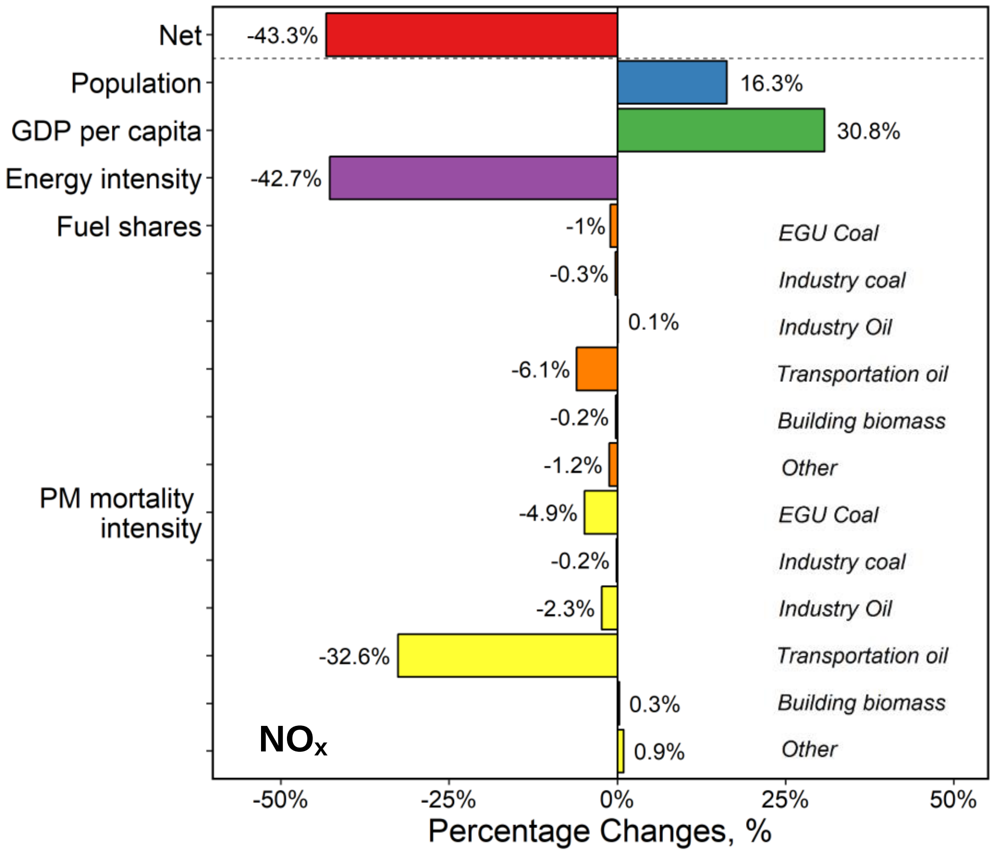

Figure S4 Net change in $PM_{2.5}$ mortality costs attributed to NO_x emissions and contributions due to changes in population, GDP per capita, energy intensity, fuel shares, and $PM_{2.5}$ mortality intensity for the contiguous U.S. in 2050 relative to 2015.

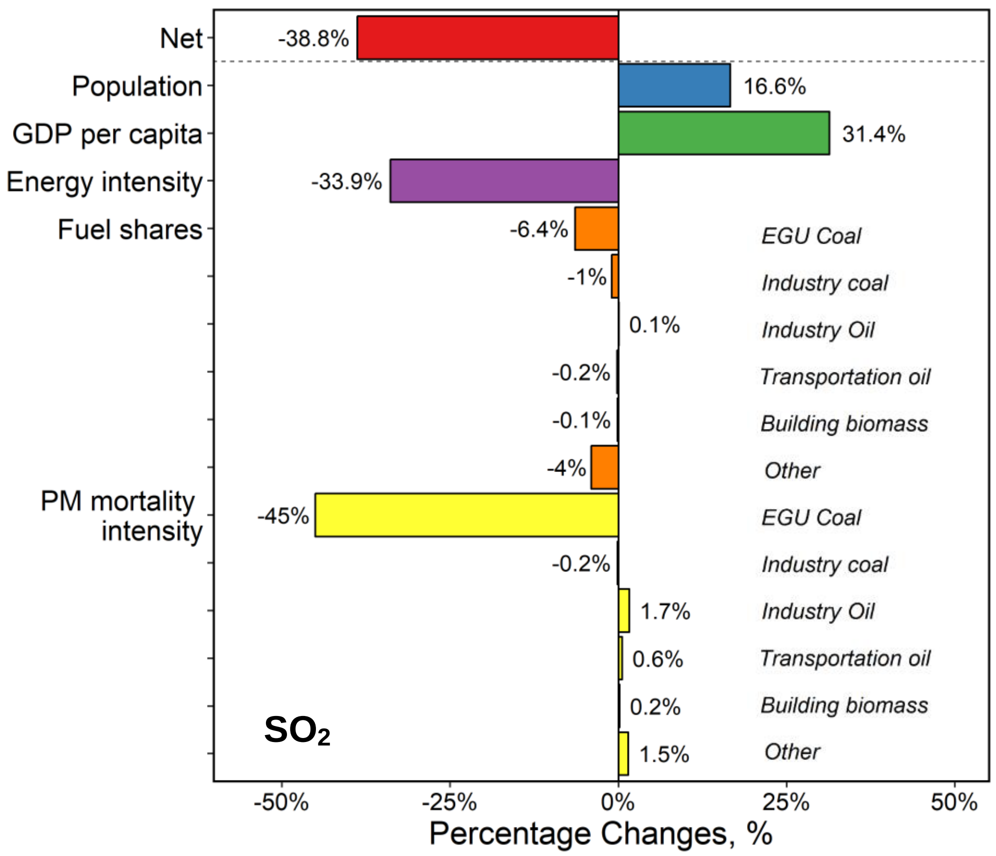

Figure S5 Net change in $PM_{2.5}$ mortality costs attributed to SO_2 emissions and contributions due to changes in population, GDP per capita, energy intensity, fuel shares, and $PM_{2.5}$ mortality intensity for the contiguous U.S. in 2050 relative to 2015.

Table S6 Abbreviations and full names of US states

Abbreviation	Full name	Abbreviation	Full name
AK	Alaska	MT	Montana
AL	Alabama	NC	North Carolina
AR	Arkansas	ND	North Dakota
AZ	Arizona	NE	Nebraska
CA	California	NH	New Hampshire
CO	Colorado	NJ	New Jersey
CT	Connecticut	NM	New Mexico
DC	District of Columbia	NV	Nevada
DE	Delaware	NY	New York
FL	Florida	OH	Ohio
GA	Georgia	OK	Oklahoma
HI	Hawaii	OR	Oregon
IA	Iowa	PA	Pennsylvania
ID	Idaho	RI	Rhode Island
IL	Illinois	SC	South Carolina
IN	Indiana	SD	South Dakota
KS	Kansas	TN	Tennessee
KY	Kentucky	TX	Texas
LA	Louisiana	UT	Utah
MA	Massachusetts	VA	Virginia
MD	Maryland	VT	Vermont
ME	Maine	WA	Washington
MI	Michigan	WI	Wisconsin
MN	Minnesota	WV	West Virginia
MO	Missouri	WY	Wyoming
MS	Mississippi		

REFERENCES

Abt Associates, Inc. Environmental Benefits Mapping and Analysis Program (BenMAP), Version 4.0.35; Prepared for Office of Air Quality Planning and Standards, U.S. Environmental Protection Agency: Research Triangle Park, NC, 2010.

Fann N, Fulcher CM, Baker K. 2013. The recent and future health burden of air pollution apportioned across U.S. sectors. Environmental Science and Technology. 47(8), 3580-3589

Heo J, Adams PJ, Gao HO. 2016a. Reduced-form modeling of public health impacts of inorganic $PM_{2.5}$ and precursor emissions. Atmospheric Environment. 137, 80-89

Heo J, Adams PJ, Gao HO. 2016b. Public health costs of primary $PM_{2.5}$ and inorganic $PM_{2.5}$ precursor emissions in the United States. Environmental Science and Technology. 50, 6061-6070

H.R.6 – Energy Independence and Security Act of 2007. 110th Congress (2007-2008)

Iyer G, Ledna C, Clarke LE., McJeon H, Edmonds J, Wise M. 2017. GCAM-USA Analysis of US Electric Power Sector Transitions, Pacific Northwest National Laboratory, Richland, Washington.

Joint Global Change Research Institute (JGCRI), (2019). GCAM v5.1 documentation: GCAM-USA. Univ. of Maryland and Pacific Northwest National Laboratory. http://jgcri.github.io/gcam-doc/gcam-usa.html (accessed Mar 5, 2019)

Krewski D, Jerrett M, Burnett RT, Ma R, Hughes E, Shi Y, Turner MC, Pope CA III, Thurston G, Calle EE, et al. Extended follow-up and spatial analysis of the American Cancer Society study linking particulate air pollution and mortality, Research Report 140; Health Effects Institute: Boston, MA, 2009.

Lepeule J, Laden F, Dockery D, Schwartz J. Chronic Exposure to Fine Particles and Mortality: An Extended Follow-up of the Harvard Six Cities Study from 1974 to 2009. Environ. Health Perspect. 2012, 120 (7), 965−970.

Muller NZ. 2014. Boosting GDP growth by accounting for the environment: Including air pollution and greenhouse gas damages increases estimated US growth. Science. 345(6199), 873-874

S&P Global Platts. 2018. No new nuclear units will be built in US due to high cost: Exelon official. Available at: https://www.spglobal.com/platts/en/market-insights/latest-news/electric-power/041218-no-new-nuclear-units-will-be-built-in-us-due-to-high-cost-exelon-official. [accessed Jan 2019]

Senate Bill No. 1368. (2006). SB 1368, Perata. Electricity: emission of greenhouse gases. Available a: https://www.energy.ca.gov/emission_standards/documents/sb_1368_bill_20060929_chaptered.pdf [accessed Jan 2019]

Shi W, Ou Y, Smith SJ, Ledna CM, Nolte CG, Loughlin DH. 2017. Projecting state-level air pollutant emissions using an integrated assessment model: GCAM-USA. Applied Energy, 208, 511-521.

Tessum CW, Hill JD, Marshall, JD. 2017. InMAP: A model for air pollution interventions. PLoS ONE 2017, 12 (4), e0176131

Union of Concerned Scientists (UCS). (2017). Going from pump to plug: Adding Up the Savings from Electric Vehicles (EVs). Available at: https://www.ucsusa.org/clean-vehicles/electric-vehicles/ev-fuel-savings [accessed Jul 2019]

US Energy Information Administration (EIA). 2018. Annual Energy Outlook 2018. Available at: https://www.eia.gov/outlooks/aeo/. [accessed Nov 2018]

US EPA. (2019). Mortality Risk Valuation. Available at: https://www.epa.gov/environmental-economics/mortality-risk-valuation. [accessed Jan 2019]

US EPA. (2015a). 40 CFR Parts 60. Standards of Performance for New Residential Wood Heaters, New Residential Hydronic Heaters and Forced-Air Furnaces. Fed. Register, 2015, Vol. 80, No. 50, 13672-13753.

US EPA. (2015b). Environmental Benefits Mapping and Analysis Program – Community Edition (BenMAP-CE) Version 1.1. US Environmnetal Protection Agceny., Research Triangle Park.

Vermont Department of Public Service (DPS), Comprehensive Energy Plan 2016, Executive Summary. Available at: https://publicservice.vermont.gov/sites/dps/files/documents/Pubs_Plans_Reports/State_Plans/Comp_Energy_Plan/2015/2016CEP_ES_Final.pdf [accessed Jan 2019]

Viscusi, WK, Aldy, JE. 2003. The value of a statistical life: A critical review of market estimates throughout the world. J. Risk and Uncertainty 27, 5-76.
